# Effect of dietary fiber intake on blood pressure control in type 2 diabetic patients in selected hospitals of Ethiopia

**DOI:** 10.1038/s41598-025-24579-3

**Published:** 2025-11-19

**Authors:** Feven Hailu, Jemal Haidar Ali, Yakob Desalegn Nigatu

**Affiliations:** Department of Nutrition and Dietetics, School of Public Health, Addis Ababa University, Addis Ababa, Ethiopia

**Keywords:** Dietary fiber intake, Hypertension, Glycemic control, Diabetes mellitus, Addis ababa, Diseases, Endocrinology, Health care, Medical research, Risk factors

## Abstract

Dietary fiber contributes to cardio metabolic health, yet contextual evidence in Ethiopia is scarce. This study examined the effect of dietary fiber intake on blood pressure (BP) control among patients with type 2 diabetes. An institutional-based cross-sectional study was conducted from March to April 2024 among 287 type 2 DM patients. Dietary intake was assessed using a repeated multiple-pass 24-hour recall, which involved collecting detailed recipes and estimating portion sizes in grams using dietary scales, proxy indicators, and standardized conversion and yield factors. Nutrient analysis, including dietary fiber (g/day), was performed using NutriSurvey 2007 based on the Ethiopian Food Composition Table and complementary regional and USDA sources. Blood pressure was measured using a calibrated digital sphygmomanometer, and HbA1c values were extracted from recent laboratory records. A significant proportion (79.4%) of respondents exhibited poor blood pressure control, while over half (56.7%) demonstrated poor glycemic control. Additionally, 10.3% of participants were classified as obese and 39% had stage 2 hypertension. The mean dietary fiber intake was 32.5 ± 20 g/day. Fiber intake showed significant inverse correlations with BP (*r* = -0.551), BMI (*r* = -0.605), and HbA1c (*r* = -0.341), all *p* < 0.001. In multivariable logistic regression, adjusted for age, duration of diabetes, diet, and physical exercise, showed that higher dietary fiber intake remained an independent predictor of good blood pressure control. Each 1 g/day increase in dietary fiber was associated with a 6% reduction in the odds of poor BP control (AOR = 0.936; 95% CI: 0.913–0.959; *p* < 0.001). Regular physical activity also significantly reduced the odds of poor BP control (AOR = 3.35 for non-exercisers). Higher dietary fiber intake and regular exercise were strongly associated with improved BP and glycemic control in type 2 diabetes patients. These findings support not only public health initiatives to promote fiber-rich diets but also integration of dietary counseling and lifestyle modification into routine clinical diabetes and hypertension management.

## Introduction

Cardiovascular diseases (CVDs) remain the leading cause of morbidity and mortality worldwide, accounting for over 20 million deaths in 2021^1^ .Although non‑modifiable factors such as age and family history contribute to risk, modifiable determinants including diet and physical activity are central to prevention and control. Contemporary guidance underscores nutrition as a cost‑effective strategy for lowering blood pressure (BP) and cardiovascular risk^2,3^.

Among lifestyle interventions, dietary fiber (DF) has attracted considerable attention. DF is a carbohydrate polymer composed of ten or more monomeric units that cannot be digested by small intestinal enzymes. It can be classified as soluble or insoluble based on solubility, viscosity, and ferment ability. Soluble fibers form viscous, fermentable gels, while insoluble fibers are water-insoluble and poorly fermentable^4^. Most natural foods contain both types of fiber, with cereals, legumes, fruits, and vegetables serving as major sources; approximately one-third of total fiber is soluble, and the remaining is insoluble^5,6^.

Accumulating evidence supports a causal role of dietary fiber in blood pressure regulation. Systematic reviews and meta-analyses indicate that higher fiber intake can reduce systolic BP by approximately 2–3 mmHg and improve glycemic control among adults with type 2 diabetes mellitus (T2DM)^2^. Mechanistically, fermentation of fiber produces short-chain fatty acids that may enhance endothelial function and arterial compliance, while fiber-rich diets improve insulin sensitivity and body weight regulation, pathways that are directly relevant to BP control^7,8^ .

Despite these findings, most existing evidence originates from Western populations, and local data in Ethiopia and Sub-Saharan Africa are scarce. Prior studies in African populations have evaluated diet–BP associations primarily among diabetics, yet none have comprehensively examined dietary fiber intake in relation to blood pressure in Ethiopian adults with T2DM^9–11^. National surveys highlight a substantial hypertension burden^9,10^, underscoring the importance of establishing context-specific evidence linking quantified fiber intake with BP outcomes using standardized dietary assessments^12,13^.Therefore, this study aimed to quantify fiber intake, identify major food sources, and examine its association with blood pressure control among adults with type 2 diabetes in Addis Ababa.

## Methods and materials

### Study design, period and participants

An institution-based cross-sectional study was conducted among Type 2 diabetes mellitus (T2DM) patients who had at least a three-month follow-up at general government hospitals in Addis Ababa, Ethiopia, from March to April 2024. The study included four general hospitals with chronic disease follow-up clinics: Zewditu Memorial Hospital (ZMH), Yekatit 12 Hospital and Medical College, Tirunesh Beijing Hospital, and Ras Desta Damtew (RDD) Hospital. These hospitals were selected because they are the only general hospitals in Addis Ababa providing comprehensive diabetes follow-up care, making them representative of public hospital-based diabetes management in the city. Referral hospitals were excluded to minimize selection bias, as the prevalence and severity of comorbidities, including advanced cardiovascular disease, tend to be higher in referral settings and could confound the relationship between dietary fiber intake and blood pressure control.

The inclusion criteria were T2DM patients aged 18 years and above with at least a three-month follow-up at the selected hospitals. Exclusion criteria included patients in acute medical distress, pregnant or lactating women, and individuals with critical illnesses such as advanced malignancy or compensated liver cirrhosis, and those undergoing renal replacement therapy.

### Sample size and sampling technique

The sample size (SS) for each specific objective was calculated individually. For the first descriptive objective, which aimed to identify the major sources of dietary fiber, the sample size was computed using a single population proportion formula. The study considered the most frequently consumed food items and their respective fiber content, including cereals (teff = 9.8%), fruits (avocado = 3.1%), vegetables (kale = 20.4%), nuts (2.8%), tubers (2.8%), seeds (sunflower = 21.7%), and legumes (chickpea = 16.9%)^14–19^.

Sunflower seeds were chosen as the reference for the calculation because they have the highest fiber proportion among commonly consumed foods in the study setting and are frequently eaten as ‘suf fitfit’—a local dish made by mixing injera with water flavored with ground sunflower seeds. Using the food item with the largest proportion is a standard approach, as it provides the largest variance and, consequently, the largest sample size, ensuring a conservative estimate. Considering a 95% confidence level, 5% level of significance, 5% margin of error, and 10% non-response rate, the calculated sample size for this objective was 287 participants.

For the second objective, which aimed to estimate fiber intake in Type 2 diabetics, a single mean formula was used. The calculation considered a mean of 19.27 ± ± 7.07^9^ a 5% level of significance, a margin of error equal to 5% of the mean, and a 10% non-response rate, resulting in a sample size of 228. Since the sample size estimated for the first objective covered all requirements, the final working sample size remained at 287 participants.

### Sampling procedure

**Fig. 1:**
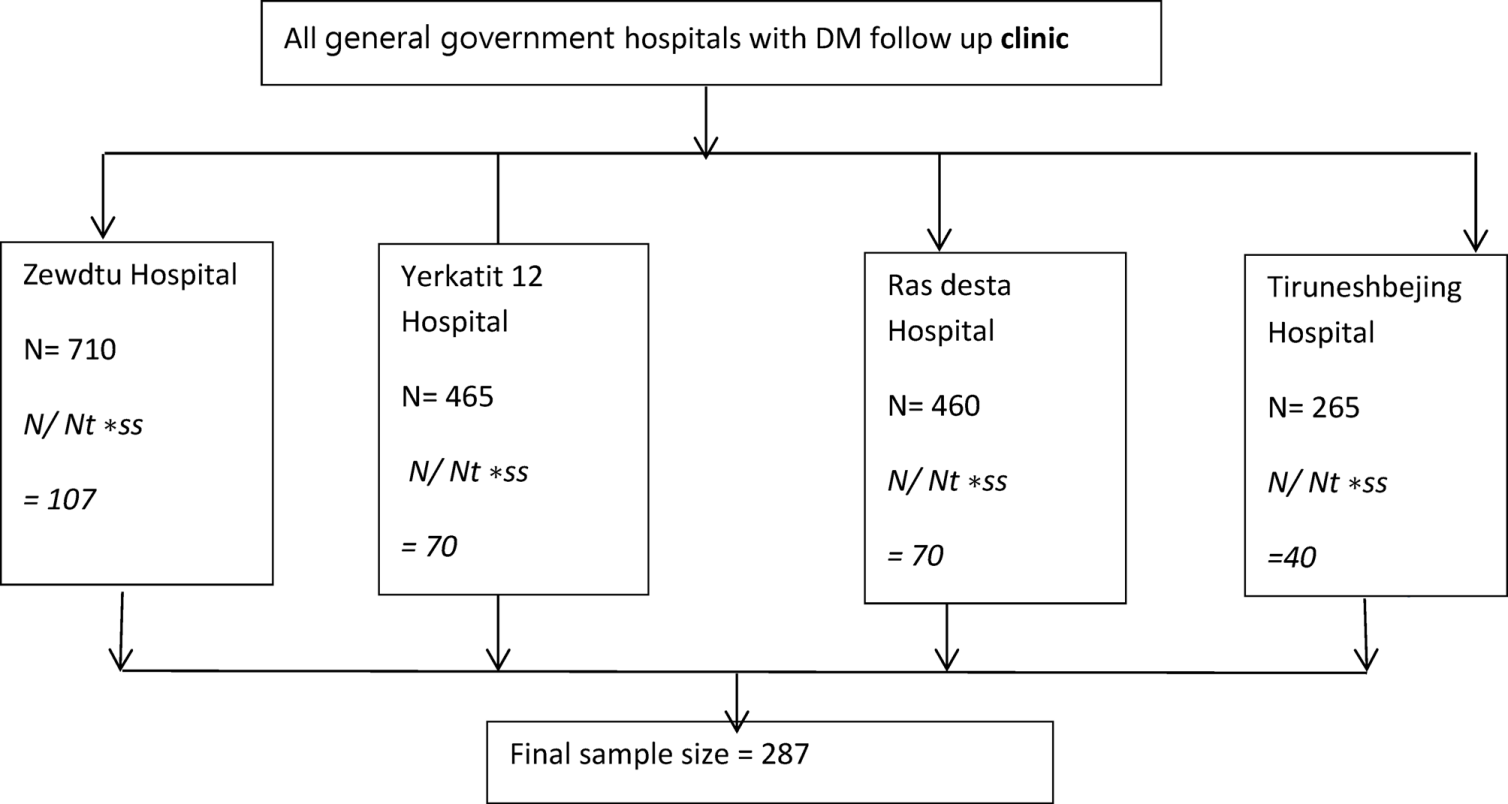
Schematic representation of sampling procedures.

The study was conducted in four government general hospitals: Zewedtu Memorial Hospital, Yekatit 12 Hospital and Medical College, Tirunesh Beijing Hospital, and Ras Desta Dametew Hospital. The three-month average patient flow at the endocrine outpatient departments was 710, 465, 460, and 265 Type 2 DM patients per month, respectively. Study participants were selected using a systematic sampling technique (N/SS = 1900/287 ≈ 7), approaching every seventh patient attending appointments until the predetermined sample size was reached. The final allocation of participants per hospital was described below (Fig. 1).

### Data collection procedures and measurements

Data collection was carried out over a month at specific government hospitals, led by a team of four trained public health officers(BSc) and two nutrition supervisors(BSc in nutrition), employing the KOBO toolbox. Prior to data collection, the principal investigator provided a two-day training session focused on the study’s goals, data collection techniques, 24-hour recall interview methods, and the operation of the KOBO toolbox.

The study utilized a structured tool to obtain data on respondents’ socio demographic information, health behaviors, and clinical profiles. The socio demographic section included inquiries about age, sex, marital status, family size, and occupation, education, and income levels. Health behaviors were assessed through questions regarding cigarette smoking, alcohol use, Chat chewing, and physical activity. Clinical details covered aspects like disease duration, treatment types, presence of comorbidities, family history of diabetes mellitus, and current medications being taken.

Weight and height were recorded using a balanced digital scale accurate to 0.1 kg, with participants wearing light clothing and no shoes. Height was measured to the nearest 0.1 cm while participants stood straight, ensuring their heads, backs, and buttocks were aligned. Body Mass Index (BMI) was calculated by dividing weight in kilograms by the square of height in meters (kg/m²), following standard protocols during interviews.

Blood pressure was assessed using a digital electronic sphygmomanometer after ensuring respondents rested for at least five minutes. Two readings were taken with a two- to three-minute interval to ensure accuracy; the average of these was recorded. If the readings differed by more than 10 mmHg, a third measurement was taken, and the average of all three was used to determine blood pressure status.

Dietary intake was assessed using a multiple-pass 24-hour recall, in which respondents reported all foods and beverages consumed, portion sizes, eating times, and locations. Recall bias was minimized through several measures implemented both prior to and during data collection. Prior to data collection, a recipe collection exercise was conducted to document commonly consumed foods, ingredients, and preparation methods in the study setting, providing interviewers with a consistent reference for probing participants. During the interviews, a four-step probing technique was employed: listing items, quantifying amounts, collecting recipes, and reviewing the recall for completeness. Portion sizes were estimated using standard units, size/number estimation, play dough for high-viscosity foods (e.g., fruits, bread), rice for medium-viscosity foods (e.g., stews), and water for liquids. Standardized portions were applied for items such as injera (310 g) and commercially available foods like beer. Rice and play dough portions were weighed using a digital dietary scale, and actual intake was calculated using appropriate conversion factors.

**Table 1:** Conversion factors for PSEM.

PSEM	Conversion factor
Rice	0.82
Play dough	1.217
Water	1
Injera	Standard one Injera = 310 g which was taken from national survey (EPHI)

Nutrient composition was primarily obtained from the Ethiopian Food Composition Table and supplemented with data from Kenya, West Africa, and the USDA; items not found in these sources were estimated proportionally.

Since a single 24-hour recall cannot capture day-to-day variation, a second non-consecutive 24-hour recall was conducted on 10% of participants (*n* = 29) during their next follow-up. A strong correlation between the first and second recalls (*r* = 0.728, *p* < 0.01) indicated that the first recall provided reliable estimates of mean intake at the group level, supporting the use of a single recall for the majority of participants.

Fasting blood glucose (FBG) levels and hemoglobin A1C data were obtained from patient records, with the latest HbA1C readings taken one to three months before data extraction.

### Operational definitions

Blood pressure was classified as normal with a reading of 120/80 mm Hg, elevated when it fell between 120 and 129/80 mm Hg, Stage 1 hypertension when systolic BP ranged from 130 to 139 mm Hg or diastolic BP was 80–89 mm Hg, and Stage 2 hypertension when systolic BP exceeded 140 mm Hg or diastolic BP reached 90 mm Hg or higher, regardless of anti-hypertensive medication use^3^. Good blood pressure was categorized below < 120/80 and above that were included as poor blood pressure control^20^ .

Good glycemic control was indicated by an HbA1c level below 7% and a fasting blood sugar level between 100 and 125 mg/dL, whereas poor glycemic control was characterized by an HbA1c above 7% and a fasting blood sugar level of 70 mg/dL or more^21^.

For individuals aged 18 to 64, adequate physical activity was considered to be a minimum of 150 to 300 min of moderate-intensity aerobic exercise per week^22^. Body weight classifications were established based on BMI: participants were categorized as underweight if their BMI was below 18.5 kg/m², normal weight if their BMI was below 25 kg/m², overweight if their BMI was between 25 and 29.9 kg/m², and obese if their BMI was 30 kg/m² or higher^23^.

At the time of the study, a person was classified as a smoker if they smoked at least one cigarette daily. Patients who consumed more than two units of alcohol per day for male patients and more than one unit of alcohol per day for female patients were classified as alcohol consumers^20^ .

### Data entry and processing

Day-to-day communication was maintained between the data collectors and the principal investigator (PI) regarding the collected and entered data. The assigned supervisor regularly shared the data with the PI to check for any missing information in a timely manner and to ensure data quality. In addition to site-based supervision, phone calls were made to data collectors to address any issues that arose during the data collection process. Once the data were collected, it was cleaned and coded before being exported to SPSS version 27 for both descriptive and inferential statistics. Before running multivariable regression, multicollinearity was assessed using Variance Inflation Factor (VIF), with all variables having VIF < 2, indicating no concerning multi-collinearity. A binary logistic regression model was employed to evaluate the determinants of blood pressure control (the outcome variable). Factors with a p-value of less than 0.25 in bi variable analyses was then included in the multivariable binary regression analysis. For all statistical significance tests, adjusted odds ratios (AOR) with a 95% confidence interval (CI) and a p-value of less than 0.05 were utilized. The results of the logistic regression indicated that the selected model had a good fit, as evidenced by the Hosmer-Lemeshow goodness-of-fit statistic of 0.712, which was greater than 0.05.

### Ethical approval and informed consent

Permissions were granted by the health facilities involved. Ethical clearance was secured from the SPH Review Board (Reference No: SRH/296/2024). Oral consent was obtained from all participants due to low literacy levels and to avoid discomfort associated with signing documents. This approach was approved by the Institutional Review Board and aligns with the Ethiopian National Research Ethics Review Guideline for minimal-risk studies. Participants were informed about the study objectives, data privacy, and confidentiality before providing consent and also participants who did not receive services in line with the standard treatment guidelines were counseled to address the discrepancies. All data collection procedures were performed in accordance with standard protocol.

## Result

### Socio-demographic characteristics of respondents

Of the 287 sampled participants, 282 (98.2%) of them had complete set of data. Less than half (44.32%) were between the ages of 50 and 65. The majority of them (72.3%) were orthodox Christians. Males made up about half (51.41%) of them. Over three-quarters (78.72%) of the respondents were married, and about half (50.35%) of them were part of households with four to six family members. About 38.29% had completed university education or higher. Regarding occupation, 38 (13.47%) were employed by non-governmental organizations, and over half (58.51%) of them made more than 5,000 birr (Table 1).

**Table 2:** Socio-demographic characteristic of respondents.

Variable	Frequency (*N*)	Percent (%)
Sex		
Male	145	51.41
Female	137	48.58
Age in years		
18–34	13	4.60
35–49	74	26.24
50–65	125	44.32
Above 65	70	24.82
Religion		
Orthodox	204	72.3
Protestant	37	13.1
Muslim	35	12.4
Others	6	2.12
Educational status		
No formal education	67	23.75
Primary school and Secondary completed	107	37.94
College or University above	108	38.29
Marital status		
Married	222	78.72
Divorced	38	13.47
Widowed	22	7.80
Family size members		
1–3	104	36.87
4–6	142	50.35
Above 7	36	12.76
Occupation		
Self-employed	86	30.49
Retired	63	22.34
Government employed	40	14.18
Non-government employed	38	13.47
House wife	33	11.70
Non employed	22	7.80
Salary (birr)		
Less than 1500	39	13.82
1500–5000	78	27.65
Above 5000	165	58.51

Other * catholic.

### Behavioral factors affecting blood pressure control

**Fig. 2:**
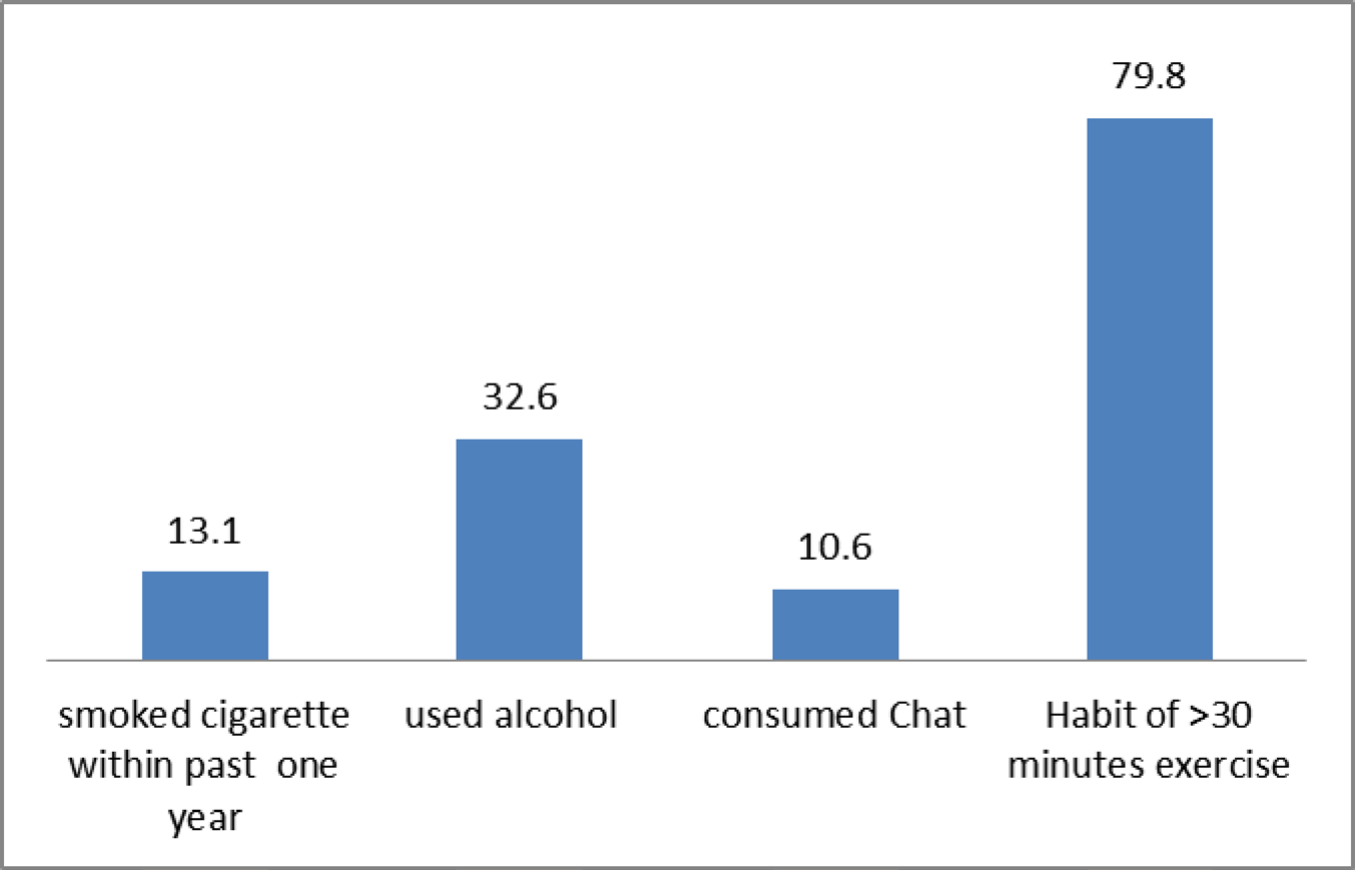
Behavioral factors affecting blood pressure control among respondents (% of respondents reporting behaviors).

As displayed in Fig. 2, the proportion of respondents who have smoked cigarettes within one year, consumed chat, alcohol, and had the habit of doing physical exercise for more than 30 min on most days of the week were 10.6%, 13.1%, 32.6%, and 79.8%, respectively.

### Clinical characteristic of the respondents

**Fig. 3:**
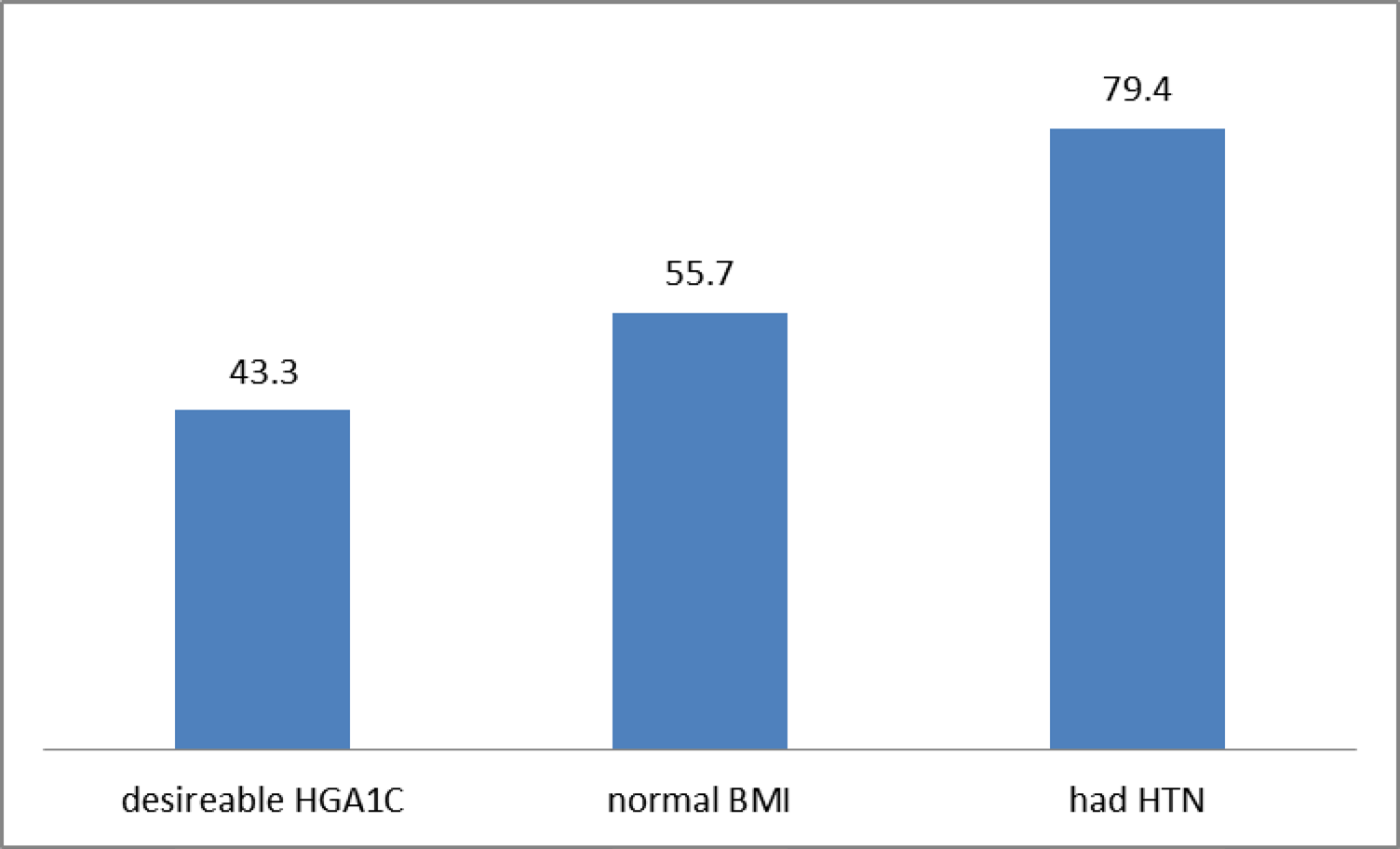
Clinical characteristics of the respondents (% of respondents).

A total of two hundred twenty-four (79.4%) of respondents had hypertension. The proportion of respondents with desirable hemoglobin A1C and normal BMI was 43.3% and 55.7%, respectively. (Fig. 3)

### Health profile of respondents

As shown in Table 2, approximately two-thirds (64.9%) of participants had been living with the disease for over five years, and more than half (56.4%) of these individuals experienced comorbidities. The most prevalent comorbidity was hypertension, affecting 99 participants (61.1%), followed by hyperlipidemia in 42 participants (25.9%) and cardiovascular disease (CVD) (7.4%). The majority of respondents were on multi-therapy for hypertension, and one-third reported a family history of the condition. Most (90.1%) made dietary modifications upon being diagnosed with both HTN and DM. Additionally, nearly half (46.1%) had a history of hospital admissions, with approximately 41.5% of these admissions attributed to complications related to HTN and DM.

**Table 3:** Health profile of the respondents.

Variables	Frequency (*n* = 282)	Percent
Duration of diabetes (years)		
Less than 5	99	35.1
5–10	99	35.1
More than 10	84	29.8
Comorbidity disease		
Yes	162	56.4
No	125	43.6
Type of comorbidity (*n* = 162)		
Hypertension	99	61.1
Hyperlipidemia	42	25.9
Cardiovascular diseases	12	7.4
Asthma	9	5.6
Method of DM control		
Insulin	50	17.7
Tablet	100	35.5
Both	132	46.8
Duration of HTN follow up (years)		
Less than 2	84	29.8
3–5	88	31.2
More than 5	110	39.0
Methods of control of HTN		
Mono-therapy (1 medication)	21	7.4
Multi -therapy (more than one)	262	92.6
Family history of HTN		
Yes	66	23.5
No	216	76.5
Dietary modification since follow up		
Yes	254	90.1
No	28	9.9
Admission history		
Yes	130	46.0
No	152	54.0
Complications related to HTN&DM		
Yes	117	41.5
No	165	58.5

### Calorie intake of respondents

Calorie intakes of respondents were analyzed using multi pass 24-hour dietary assessment method. The estimated mean energy intake based on the 24 h. recall assessment was 1673.95 ± 743.1. Carbohydrate accounted for 73.1% of the total energy consumed, and fat contributed 14.5% and the rest 12.3% of the total energy was from protein (Tables 3 and 4).

**Table 4:** Calorie intake of respondents vs recommended.

Energy sources	Calorie (mean + SD )	Percent contribution to total calorie (100%)	Recommended daily allowance (RDA)
Energy (kcal)	1673.95 ± 743.1	100	2000–3000 kcal/day
Carbohydrate (g/day)	295.86 ± 124.9	73.1	50–60 (% of total energy)
Protein (g/day)	50 ± 24.9	12.3	10–15% (% of total energy)
Fat (g/day)	28.12 ± 26	14.5	25–30% (% of total energy)

### Fiber intake and major sources of fiber

**Fig. 4:**
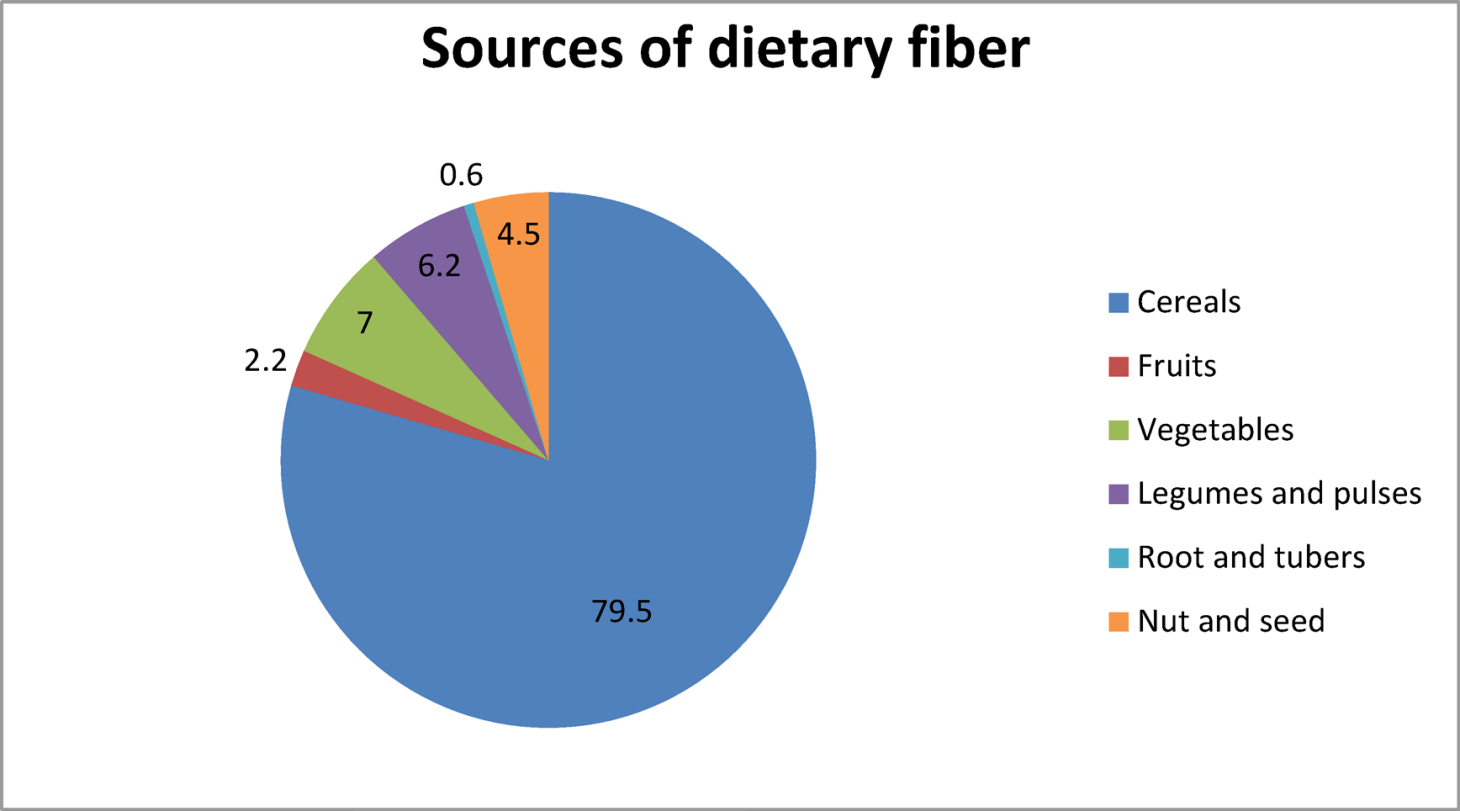
Major food sources of dietary fiber among respondents (% contribution to total dietary fiber intake).

The mean dietary fiber intake was 32.5 ± 20 g/d. From the foods, six groups of food types were identified as the major sources of dietary fiber. The leading source was cereals, which contributed 79.5% of the total fiber intake, followed by vegetables (7%), legumes and pulses (6.2%), and nuts and seeds (4.5%). The predominance of cereals was notable, indicating that the bulk of fiber intake among participants came from cereal-based staples, while other food groups such as vegetables, legumes, and fruits made relatively smaller contributions (Fig. 4).

### Correlation between fiber intake and various health indicators

We utilized the Pearson correlation coefficient to investigate the association between dietary fiber intake and various health indicators. The results showed a significant inverse correlation between dietary fiber intake and both blood pressure (coefficient: -0.551, p-value < 0.001) as well as body mass index (coefficient: -0.605, p-value < 0.001). This suggests that higher dietary fiber intake was linked to lower levels of blood pressure and body mass index. Additionally, the analysis also revealed a significant inverse correlation between dietary fiber intake and hemoglobin A1c (coefficient: -0.341, p-value < 0.001), indicating that increased dietary fiber intake is associated with lower hemoglobin A1c levels (Table 5).

We utilized the Pearson correlation coefficient to investigate the association between dietary fiber intake and various health indicators. The results showed a significant inverse correlation between dietary fiber intake and both blood pressure (coefficient: − 0.551, *p* < 0.001) and body mass index (coefficient: − 0.605, *p* < 0.001), indicating that higher dietary fiber intake was linked to lower levels of blood pressure and body mass index. Similarly, there was a significant inverse correlation between dietary fiber intake and hemoglobin A1c (coefficient: − 0.341, *p* < 0.001), suggesting that increased dietary fiber intake was associated with better glycemic control.

**Table 5:** Correlation between fiber intake and various health indicators among respondents.

Correlations		dietary fiber ( g)	Body mass index	Blood pressure	hemoglobinA1Cnew
Dietary fiber ( g)	Pearson correlation	1	− 0.605^**^	− 0.551^**^	− 0.341^**^
	P value		0.000	0.000	0.000
	N	282	282	282	268

**Correlation is significant at the 0.01 level (2-tailed).

### Subgroup analysis of blood pressure control

Table 6 outlined subgroup differences in blood pressure control according to sex, age, and comorbidity status. No statistically significant difference was observed between men and women (*p* = 0.218). However, age was significantly associated with BP control (*p* = 0.006), with younger participants (18–34 years) showing better control compared to older age groups. Regarding income, the proportion of good BP control was relatively higher among participants in the higher salary group (58.6%) compared to the lower salary groups. Nevertheless, poor BP control remained common across all income categories, including those with higher salary.

Comorbidity was also significantly associated with BP control (*p* = 0.040), with those having comorbid diseases being more likely to have uncontrolled blood pressure compared to participants without comorbid conditions (Table 6).

**Table 6:** Subgroup analysis of blood pressure control by age, sex and comorbidity.

Variable	Blood pressure control		*P* value
	Poor BP control n (%)	Good BP control n (%)	
Sex			
Male	111 (49.6%)	34 (58.6%)	0.218
Female	113 (50.4%)	24 (41.4%)	
Age			
18–34	6 (2.7%)	7 (12.1%)	0.006
35–49	58 (25.9%)	16 (27.6%)	
50–65	98 (53.8%)	27 (46.6%)	
Above 65	62 (27.7%)	8 (13.8%)	
Income (birr)			0.015
Less than 1500	35 (15.6%)	4 (6.9%)	
1500–5000	58 (25.9%)	20 (34.6%)	
Above 5000	131 (58.5%)	34 (58.6%)	
Comorbidity disease			
Yes	99 (44.2%)	17 (29.3%)	0.040
No	125 (55.8%)	41 (70.7%)	

### Determinants of blood pressure control among respondents

Binary logistic regression was used to identify determinants of blood pressure control. Candidate variables with a p-value < 0.25 in the bivariate analysis (exercise, dietary fiber, energy, protein, and type of diabetic control) were entered into the multivariable model, which was further adjusted for age, type of medication, and duration of illness. After adjustment, only exercise and dietary fiber remained significant independent predictors of blood pressure control (*p* < 0.05).

Lack of physical exercise showed a positive association with poor blood pressure control, as patients who did not exercise had over threefold higher odds of uncontrolled blood pressure **(AOR = 3.35; 95% CI: 1.21–9.24)** compared to those who exercised for at least 30 min, 3–5 times per week. In contrast, dietary fiber intake demonstrated an inverse association: each unit increase in fiber intake was associated with a 6% reduction in the odds of poor blood pressure control **(AOR = 0.94; 95% CI: 0.91–0.96)** (Table 7).

**Table 7:** Bi variable and Multi-variable analysis for determining predictors of poor BP control respondents.

Variable	Category	AOR (95% CI)	*p*-value
Diabetic control	Tablet	0.69 (0.26–1.84)	0.449
	Insulin	0.51 (0.24–1.11)	0.090
	Both (ref)	1.00	–
Age	18–34	0.111(0.280–1.084)	0.180
	35–49	0.464(0.186–1.175)	
	50–65	0.468(0.200-1.096)	
	> 65(ref)	1.00	
Exercise	No	3.35 (1.21–9.24)	0.020*
	Yes (ref)	1.00	–
Energy intake	–	1.00 (0.999–1.001)	0.561
Dietary fiber	–	0.94 (0.91–0.96)	0.001*
Protein intake	–	0.99 (0.97–1.01)	0.369

1 = indicate the reference category * significance association at P value < 0.005 *Adjusted for energy, type of diabetic control, age, protein, dietary fiber and exercise.

## Discussion

This study provides new evidence from Ethiopia, showing that uncontrolled blood pressure remains highly prevalent among patients with type 2 diabetes. Importantly, lifestyle behaviors such as dietary fiber intake and physical activity were identified as independent determinants of blood pressure control. We observed that every incremental increase in fiber intake translated into a significant reduction in the odds of poor control, while absence of regular exercise markedly increased risk. These results highlight that, beyond pharmacological treatment, everyday lifestyle choices are central to achieving optimal cardiovascular outcomes in this population.

Dietary fiber in addition to its role in managing blood pressure, it also plays a vital role in blood sugar control. Those patients who consumed an average of 32.5 ± 20 g/day of DF, aligning with the recommended intake of 20–35 g/day had better control of their blood sugar and blood pressure in our study. This amount compared with other African countries, our finding was higher. For instance DF consumption was 23.2 ± 8.0 g/day in Uganda^11^, 19.73 ± 8.82 g/day for South Africa^10^, and 6.75 ± 2.83 g/day) in Sudan^11^. In contrast DF intake was slightly lower than the Kenyan patients (37.9 g/day)^24^. The higher fiber consumption in Ethiopia may be attributed to the traditional diet, which includes a variety of whole grains, legumes, and vegetables. Cultural practices and food availability also contribute to this increased intake. Conversely, the lower fiber intake compared to Kenya could result from differences in culinary traditions and agricultural practices.

When placed to broader internal context, our finding exceeds fiber intake level in East Mediterranean region (21.8 g/day) (35) and Mexico (13.83 ± 0.4 g/day) (51), Japan (8.7–21.6 g/day) (54), and Thailand (8 ± 4 g/day) (56).Such differences likely arise from variations in diet diversity and reliance on refined versus whole-grain cereals.

The prevalence of inadequate blood pressure control among respondents in this study was alarmingly high with over 39% of them classified as having severed hypertension. In contrast, a cross-sectional study conducted in the UK reported a hypertension prevalence of only 55.8%^25^, highlighting a significant disparity (55.8% vs. 79.4%) probably attributed to comorbidities such as diabetes in our study. Close to our findings, some previous studies also documented higher proportion of hypertension among urban population with the highest hit in Addis Ababa^11,20^ suggesting that individuals in this setting face additional challenges due to comorbidities that included diabetes and other cardiovascular diseases (CVDs). It is therefore crucial to emphasize the use of DF and adherence to nutritional guidelines as adjuvant approach particularly for those with multiple comorbidities.

Interestingly, our findings showed a significant correlation between blood pressure control and dietary fiber intake (*p* < 0.001). As dietary fiber intake increases, blood pressure control has improved i.e. for every unit increase in dietary fiber consumption, there was a 6% improvement in the odds of poor blood pressure control (AOR: 0.936; 95% CI: 0.913, 0.959) implying that adequate dietary fiber intake positively influences blood pressure regulation. Our finding aligns with the meta-analyses of clinical trials which demonstrated that DF intake to improve blood pressure, glycemic control, reduce insulin resistance, and promote weight loss^26^. These effects were attributed to the hypolipidemic effect of fibers which improves the elasticity of blood vessels in addition to the impact of fiber on arterial blood pressure, DF enhances insulin sensitivity and improves vascular endothelial function, both of which are crucial for regulating blood pressure^7^.

Another important finding of our study was the link of physical activity intake with poor blood pressure control, and observed to be consistent with the meta-analysis that documented performing physical activity reduces blood pressure among adults with hypertension. This is likely because physical activity decreases blood pressure through a reduction in systemic vascular resistance, involving both the sympathetic nervous system and the renin-angiotensin system, while also favorably affecting concomitant cardiovascular risk factors^27^. In addition, regular physical activity is shown to reduce blood pressure by decreasing sympathetic nerve activity in individuals with hypertension since it leads to a decreased release of norepinephrine, which is responsible for vasoconstriction, thereby lowering vascular resistance. Furthermore, physical activity enhances insulin sensitivity and diminishes insulin-related sympathetic activity. It also lessens the vascular response to endothelin-1, another vasoconstrictor affecting those with hypertension and promotes vascular remodeling, which includes the formation of new arteries, an increase in the cross-sectional area, and the enlargement of existing veins and arteries. These structural changes ultimately contribute to reduced peripheral resistance and results in reducing blood pressure^28,29^. Our findings underscore the need to integrate dietary counseling and promotion of locally available fiber-rich foods, along with physical activity, into routine diabetes and hypertension care. Community-based strategies could further reinforce behavior change and address barriers to healthy lifestyles.

Despite its strengths, this study has several limitations. First, the cross-sectional design precludes causal inference, and reverse causality cannot be ruled out (e.g., patients diagnosed with hypertension may have increased fiber intake intentionally). Second, for some participants, data collection coincided with fasting periods, which may have influenced dietary reports; however, this was mitigated by conducting a second 24-hour recall on non-fasting weekdays for a subset of participants. Moreover, in our setting, many patients with comorbidities such as diabetes and hypertension are exempted from fasting, which likely reduced the overall impact of fasting on dietary intake. Third, although we adjusted for type of medication, we did not have detailed data on adherence to antihypertensive or hypoglycemic drugs, which could confound the observed associations. Finally, as cereals accounted for nearly 80% of total fiber intake, the findings largely reflect Ethiopia’s staple-based dietary pattern. While this strengthens the internal validity of our findings for Ethiopia and similar populations, it may limit generalizability to settings with more diverse diet.

## Conclusion

Our findings highlight the urgent need to strengthen diabetes management by incorporating dietary fiber promotion and physical activity into standard care. Routine nutrition counseling in clinics should emphasize affordable, locally available fiber-rich foods, while community-based programs can reinforce behavior change through education and peer support. For patients with multiple comorbidities, multidisciplinary care models are essential. Policymakers should prioritize integrating these strategies into national diabetes and hypertension guidelines to improve long-term outcomes.

## Data Availability

The datasets used and/or analyzed during the current study available from the corresponding author on reasonable request.

## Abbreviations

AAU: Addis Ababa University
ADA: American Diabetes Association
BMI: Body mass index
BP: Blood pressure
CVD: Cardio vascular diseases
DM: Diabetes mellitus
DF: Dietary fiber
DBP: Diastolic blood pressure
FBS: Fasting blood sugar
HbA1C: Hemoglobin A1C
NCD: Non communicable disease
PSEM: Portion size estimation method
PPG: Postprandial glucose
RDA: Recommended daily allowance
SPSS: Statistical product and service solutions
SS: Sample size
SBP: Systolic blood pressure
WHO: World Health Organization
